# A Dense Linkage Map for Chinook salmon (*Oncorhynchus tshawytscha*) Reveals Variable Chromosomal Divergence After an Ancestral Whole Genome Duplication Event

**DOI:** 10.1534/g3.113.009316

**Published:** 2013-12-30

**Authors:** Marine S. O. Brieuc, Charles D. Waters, James E. Seeb, Kerry A. Naish

**Affiliations:** School of Aquatic and Fishery Sciences, University of Washington, Seattle, Washington 98195-5020

**Keywords:** whole genome duplication, chromosome homeologies, linkage map, Chinook salmon, RAD sequencing

## Abstract

Comparisons between the genomes of salmon species reveal that they underwent extensive chromosomal rearrangements following whole genome duplication that occurred in their lineage 58−63 million years ago. Extant salmonids are diploid, but occasional pairing between homeologous chromosomes exists in males. The consequences of re-diploidization can be characterized by mapping the position of duplicated loci in such species. Linkage maps are also a valuable tool for genome-wide applications such as genome-wide association studies, quantitative trait loci mapping or genome scans. Here, we investigated chromosomal evolution in Chinook salmon (*Oncorhynchus tshawytscha*) after genome duplication by mapping 7146 restriction-site associated DNA loci in gynogenetic haploid, gynogenetic diploid, and diploid crosses. In the process, we developed a reference database of restriction-site associated DNA loci for Chinook salmon comprising 48528 non-duplicated loci and 6409 known duplicated loci, which will facilitate locus identification and data sharing. We created a very dense linkage map anchored to all 34 chromosomes for the species, and all arms were identified through centromere mapping. The map positions of 799 duplicated loci revealed that homeologous pairs have diverged at different rates following whole genome duplication, and that degree of differentiation along arms was variable. Many of the homeologous pairs with high numbers of duplicated markers appear conserved with other salmon species, suggesting that retention of conserved homeologous pairing in some arms preceded species divergence. As chromosome arms are highly conserved across species, the major resources developed for Chinook salmon in this study are also relevant for other related species.

Understanding the stabilization and fate of the polyploid genome following whole genome duplication (WGD) is fundamental to evolutionary analyses (Wolfe 2001; Soltis *et al.* 2010; Mayfield-Jones *et al.* 2013). While genome duplication immediately introduces additional genetic material that can serve as a template for evolutionary innovation, the effect of this variation on adaptation and divergence rates in descendent lineages is debated (Taylor *et al.* 2001; Donoghue and Purnell 2005; Hufton and Panopoulou 2009; Mayrose *et al.* 2011). Recent duplication is pervasive in plants (Soltis *et al.* 2010) and some animals (Mable *et al.* 2011), but diversification rates in neopolyploids have been shown to be lower than that of related diploids (Mayrose *et al.* 2011). Nevertheless, comparative analyses in ancient polyploids (Ohno 1999; Lynch and Conery 2000; Canestroa *et al.* 2013) have revealed a recurring role for WGD in the evolution of eukaryote genomes. Studying the genomes of mesopolyploids-“caught in the act” of diploidization (Mayfield-Jones *et al.* 2013)-can provide a basis for understanding the processes governing genome stabilization and the persistence of duplicated regions.

Diploidization can be achieved through chromosomal rearrangements or losses, as well as through sequence deletions and divergence (Hufton and Panopoulou 2009). These processes can act together to reduce the similarity between homeologous chromosomes (ohnologs), resulting in a gradual change from multisomic inheritance based on multivalent formations at meiosis to bivalent formations and disomic inheritance at two diverged loci (Wolfe 2001). The rate of diploidization is predicted to differ between allopolyploids and autopolyploids, because the changes induced by the latter are likely to be less dramatic (Sèmon and Wolfe 2007; Doyle *et al.* 2008). Allopolyploids may attain diploid segregation earlier because the two original genomes are divergent, and thus retain progenitor contributions for longer. On the other hand, the genomes in autopolyploids are more compatible and are expected to display tetrasomic inheritance from the outset, losing parental alleles primarily through segregation. Thus, selection for diploidization may be lower in autopolyploids than in allopolyploids. One way to shed light on the processes of diploidization would be to compare the genomes of intermediate mesopolyploids that are descended from the same WGD event (Mayfield-Jones *et al.* 2013). Examining the relationship between chromosomal rearrangements and the distribution of duplicated loci in these lineages would reveal whether diploidization is uniform across the genome and between species.

It is widely accepted that a third round (3R) of genome duplication occurred in the ray-finned fish lineage after two rounds (2R) of duplication in early vertebrates (Postlethwait 2007). Salmonid fish are descended from an autopolyploid ancestor that underwent an additional (4R) event (Allendorf and Thorgaard 1984), recently estimated as occurring 58−63 million years ago (Crête-Lafrenière *et al.* 2012). Partial diploidy has been restored in this lineage through chromosomal rearrangements and divergence of homeologous chromosomes. Chromosomes have evolved by inversions within the subfamily Thymallinae, whereas chromosome structure within the subfamilies Salmoninae (which includes *Salmo*, *Salvelinus* and *Oncorhynchus*) and Coregoninae has evolved through Robertsonian rearrangements (Ohno 1970; Phillips and Rab 2001). Extensive chromosomal studies and genome mapping efforts have shown that most chromosome arms are syntenic between species (Danzmann *et al.* 2005; Phillips *et al.* 2009; Lubieniecki *et al.* 2010; Lien *et al.* 2011; Timusk *et al.* 2011; Guyomard *et al.* 2012). The chromosome arm number (NF) has been conserved (around 100) within the Salmoninae subfamily, the exception being Atlantic salmon (*Salmo salar*) which has NF = 54−58 (Allendorf and Thorgaard 1984; Phillips and Rab 2001). However, the number of chromosomes varies extensively between species, from 2n = 52−54 in Pink salmon (*O. gorbutscha*) to 2n = 84−86 in the Japanese char (*Salvelinus pluvius*), a result of differences in arm arrangements.

Evidence of tetrasomic inheritance in Salmoninae supports the fact that restoration of diploidy is incomplete in this lineage (Wright *et al.* 1983; Allendorf and Thorgaard 1984; Allendorf and Danzmann 1997). A model of secondary tetrasomy has been proposed, in which homologous chromosomes first pair in regions that are proximal to the centromere, followed by pairing between homeologs and recombination in the distal regions (Wright *et al.* 1983; Allendorf and Thorgaard 1984). Such pairing results in a greater retention of duplicated loci in regions of the chromosome involved in ongoing recombination. Occasional homeologous pairing can also result in pseudolinkage (Wright *et al.* 1983; Allendorf and Thorgaard 1984), characterized by an excess of nonparental progeny types in crosses and identified by observing linkage disequilibrium between physically unlinked loci using two-point linkage analysis (*e.g.*, Lien *et al.* 2011). In salmon species, homeologous pairing is thought to be limited to males (Wright *et al.* 1983; Allendorf and Thorgaard 1984), with only a few instances inferred in females (Danzmann *et al.* 2005; Lien *et al.* 2011). A recent linkage map based on single-nucleotide polymorphisms (SNPs) showed that duplicated loci were not randomly distributed among all chromosomes within Atlantic salmon (Lien *et al.* 2011), suggesting that diploidization rates have not been uniform among homeologous pairs. However, the distribution of duplicated loci along each homeologous chromosome pair has yet to be described. Such information in another salmon species will reveal the rates of divergence along the chromosome, and a comparative analysis will identify whether chromosomal divergence is conserved across species descended from the WGD event.

Linkage maps can facilitate genome-wide studies on diploidization that rely on chromosomal position, especially in species whose genomes have not been extensively described. Among salmonids, one such species is Chinook salmon (*O. tshawytscha*). The characterization of its genome will provide a useful comparison with the better described rainbow trout *O. mykiss* (Guyomard *et al.* 2012; Miller *et al.* 2012) and Atlantic salmon (Lien *et al.* 2011). An existing linkage map comprising 361 microsatellite markers (Naish *et al.* 2013) has been aligned to the 34 chromosomes described in Chinook salmon (Phillips *et al.* 2013). Comparative mapping using markers conserved between Chinook salmon and rainbow trout revealed that Robertsonian arm arrangements for 13 chromosomes preceded species divergence within the genus *Oncorhynchus*. An additional comparison with Atlantic salmon points to two conserved arm arrangements ancestral to the divergence of *Oncorhynchus* and *Salmo*. Since most Robertsonian fusions occur at the centromere (Slijepcevic 1998), determining the positions of the centromeres and increasing the numbers of markers on the Chinook salmon map will strengthen these comparisons and will facilitate an examination of divergence between homeologous arms. Centromere mapping has successfully been implemented in other salmonids (Thorgaard *et al.* 1983; Allendorf *et al.* 1986; Lindner *et al.* 2000; Guyomard *et al.* 2006) and is achieved by studying marker inheritance in gynogenetic diploid crosses (Komen and Thorgaard 2007). Examining the genomic distribution of markers that are recently diverged or are inherited tetrasomically can be challenging in diploids because the alleles at duplicated loci might not be fixed. However, the use of gynogenetic haploids solves this problem because offspring will only be heterozygous at duplicated loci.

The recent and rapid improvement in sequencing technologies (Hudson 2008; Shendure and Ji 2008; Metzker 2010) has facilitated the characterization of thousands of variable markers for species, or species with little or no available genetic information (Davey *et al.* 2011), such as Chinook salmon. Several of these approaches take advantage of the large amount of information afforded by sequencing a reduced portion of the genome. Restriction-site associated DNA (RAD) sequencing (Baird *et al.* 2008; c.f. Miller *et al.* 2007) targets a consistent portion of the genome across individuals. Interest for RAD sequencing has increased recently for salmon research (*e.g.*, Amish *et al.* 2012; Hecht *et al.* 2012; Houston *et al.* 2012) and is expected to provide a useful basis for comparative mapping across salmon species. Application of RAD sequencing to genome mapping in Chinook salmon provides an additional opportunity to develop analytical approaches relevant to mapping a species with polyploidy ancestry. The reliable assignment of short reads (60−100 nucleotides, typical of RAD sequencing) to loci that correspond across individuals could be resolved with the creation of a reference database of RAD loci for the species of interest, where duplicated loci would be identified. This database would rapidly facilitate alignment of newly sequenced individuals in related studies, and promote data sharing across research groups.

Our overall aims are to describe the divergence of homeologous chromosome arms in Chinook salmon following a WGD and to compare these findings with those of other salmon to determine whether the processes of diploidization are consistent across species. Our specific objectives are first to construct a reference database of RAD sequences that can be used for alignments of sequences generated in future projects. Second, we will improve the genomic map for Chinook salmon by populating the existing genome map with thousands of RAD markers from the reference database and identify chromosome arms by mapping centromere locations. Third, we will examine divergence of homologous arms by mapping duplicated loci. Finally, we will improve our current understanding of chromosome arm rearrangement between Chinook salmon and rainbow trout using comparative analyses of marker-dense maps for the two species.

## Methods

### Sample collection and creation of mapping families

We used four data sets to develop genomic resources for Chinook salmon; RAD sequences from individuals sampled across a broad geographic range for the reference database, gynogenetic haploid crosses for mapping single and duplicated loci, gynogenetic diploid crosses for placement of the centromere on linkage groups, and a diploid cross for aligning the RAD-based map with the previously identified chromosome arms (Naish *et al.* 2013; Phillips *et al.* 2013).

First, 159 individuals from a total of 10 populations from the Columbia River basin, Pacific Northwest, USA were sampled for the preliminary identification of RAD loci and creation of a reference database. Second, three haploid crosses comprising 46, 48, and 72 individuals per family were used to identify duplicated loci in the database and construct the initial linkage map. In haploid offspring, all unique loci will be homozygous; polymorphic duplicated loci will be heterozygous. The haploid crosses were created at the Cle Elum Supplementation and Research Facility by fertilizing eggs with ultraviolet-irradiated milt following the protocol of Thorgaard *et al.* (1983) and sampled before hatching. Whole embryos were collected and stored in ethanol. Third, we used three gynogenetic diploid families, created at the University of Washington hatchery facility, to map the centromere on each linkage group. The second polar body is retained during the creation of gynogenetic diploid progeny. Therefore, a progeny will be heterozygous at a locus if a crossover event occurred in the female parent between a given marker and the centromere during meiosis I. The percentage of heterozygous offspring at a locus is expected to be 0% at the centromere, increasing to 100% in the telomeric region, because salmonids exhibit complete to near complete interference and typically have one crossover event per chromosome arm (Thorgaard *et al.* 1983). Eggs were fertilized with ultraviolet-irradiated milt and subsequently heat shocked to retain the second polar body (Thorgaard *et al.* 1983). Fish were harvested as parr and stored in 100% ethanol. We sampled the dam and 84, 90 and 93 progeny from each gynogenetic diploid family. Finally, we sampled 44 F2 progeny from the diploid cross of Naish *et al.* (2013) to verify linkage group and chromosomal representation based on the microsatellite markers mapped previously, and to align the maps with the 34 chromosomes identified in Phillips *et al.* (2013). Recombination rates vary between the sexes in salmonids (Moen *et al.* 2004; McClelland and Naish 2008; Moen *et al.* 2008; Lien *et al.* 2011) we therefore mapped the female meiosis in the diploid cross to obtain accurate marker order.

### DNA extraction and sequencing

Genomic DNA was extracted using the DNeasy extraction kit (QIAGEN, Valencia, CA) following the manufacturer’s instructions. Each sample was prepared for RAD sequencing, using *Sbf*I as a restriction enzyme and six-nucleotide individual-specific barcodes, as described in Baird *et al.* (2008). Twelve to 48 individuals were then sequenced per lane on an Illumina platform (GAII or HiSeq) using 100-nucleotide single-read sequencing. Reads were sorted per individual and barcodes were removed using process_radtags implemented in Stacks (Catchen *et al.* 2011). The last 20 nucleotides were subsequently trimmed because the last 20 base pairs of the sequence had a consistently lower quality. For the purpose of this study, we defined a locus as a 74-nucleotide RAD sequence.

### Reference database of RAD loci

Creation of the reference database of RAD loci was carried out using three steps: the generation of a preliminary database of loci for Chinook salmon, the screening of the preliminary database for loci in repetitive regions and loci with repeat sequences, and the identification of duplicated loci.

Reads for all diploid individuals were sorted into polymorphic and monomorphic loci *de novo* using Stacks 0.9995 (Catchen *et al.* 2011) with a minimum of three nucleotide mismatches between loci within an individual. We retained a consensus sequence for every locus that had been sequenced with a depth greater than 5X in more than 135 individuals (85%) as a temporary database of loci: these loci were used for further screening.

The screening steps were aimed at identifying repetitive loci and loci with tandem repeat units, such as microsatellites and minisatellites. We used two alignment-based strategies. First, loci within the temporary database were aligned against themselves using Bowtie 0.12.9 (Langmead *et al.* 2009), allowing up to three nucleotide mismatches. We expect that most homeologous loci with three or fewer mismatches between the paralogs would have been identified as a single locus during the creation of the preliminary database. Paralogs with more than three mismatches would not be detected using the Bowtie alignment criteria we used here. Therefore a locus that aligned to several loci, including itself, was likely a repeat sequence and was excluded from the database. Second, a Blast search (Basic Local Alignment Search Tool; Altschul *et al.* 1990) of the temporary database was conducted against itself using the low-complexity filter implemented in the search algorithm. This filter masks regions of low complexity, such as repeat nucleotides or motifs, within the query sequence. When this filter is used, a Blast search that compares sequences with low complexity with themselves will rarely return a match or might return a match with another sequence, because the flanking sequence will be short. Therefore, loci within the database that did not return a match or where the best match (E-value less than 10^−15^) for a given locus was not itself were discarded from the temporary database.

Finally, polymorphic duplicated loci were identified using the three haploid families. Reads for all the haploid individuals were sorted into loci by alignment to the temporary database using Bowtie. Individual reads from the haploids that aligned to more than one locus in the database could not be confidently relied upon in further analyses, and so were removed from the database. Loci with a depth of less than 10 reads for an individual were discarded for that individual. Genotypes for each individual were obtained using Stacks, which uses a maximum likelihood approach to identify polymorphisms (Catchen *et al.* 2011). The presence of a single individual with a heterozygous genotype at a locus within each haploid family was considered as insufficient evidence for duplication, as this genotype could be the result of a potential sequencing error, and the locus was retained in the database. However, if more than one haploid individual was heterozygous at a locus, this locus was identified as being duplicated, since the same error occurring in two individuals was viewed as unlikely. We did not weigh these choices by family sizes, because the recurrence of a heterozygote genotype caused by sequencing error was deemed unlikely, regardless of number of offspring. This final step provided the final database of RAD loci for Chinook salmon, against which all further alignments were made.

### Linkage mapping

#### Genotyping:

Genotypes at every non-duplicated polymorphic locus in the haploid crosses were identified during the creation of the reference database. Duplicated markers identified in the haploids (Table 1) during database development were used for mapping when one of the paralogs was polymorphic (one paralog polymorphic, parental genotype aa and ab, or aa and bc) or when both paralogs were polymorphic for different alleles (both paralogs polymorphic, parental genotype ab and ac and ab and cd). We also observed loci with ab and ab parental genotypes, but did not map these loci because heterozygous offspring were uninformative.

**Table 1:** Types of duplicated loci encountered in this study, expected segregation ratio per paralog, and expected segregation ratio when both paralogs are analyzed as a single locus, which is the case in this study

Parental Genotype		Segregation Ratio Expected for Each Paralog in a Haploid Cross		Segregation Ratio Expected in the Offspring for a Haploid	Marker(s) Mapped in This Study
Paralog 1	Paralog 2	Paralog 1	Paralog 2		
aa	bb	all a	all b	all ab	None
ab	ab	0.5 a; 0.5 b	0.5 a; 0.5 b	0.25 aa; 0.5 ab; 0.25 bb	None
aa	ab	all a	0.5 a; 0.5 b	0.5 aa; 0.5 ab	Paralog 2
aa	bc	all a	0.5 b; 0.5 c	0.5 ab; 0.5ac	Paralog 2
ab	ac	0.5 a; 0.5 b	0.5 a; 0.5 c	0.25 aa; 0.25 ac; 0.25 ab; 0.25 bc	Paralogs 1 and 2
ab	cd	0.5 a; 0.5 b	0.5 c; 0.5 d	0.25 ac; 0.25 ad; 0.25 bc; 0.25 bd	Paralogs 1 and 2

The type of duplicated marker was inferred from the observed segregation ratio and the alleles observed in the offspring generation.

All the reads for the diploid cross and gynogenetic diploid crosses were aligned to the reference database using Bowtie, and the polymorphic loci were identified with Stacks. Stacks uses a maximum likelihood approach to determine whether a polymorphism in an individual is true, or whether it is due to a sequencing error (Hohenlohe *et al.* 2010). This approach can be biased against the designation of heterozygous genotypes for individuals that differ in sequence depth between the two alleles. To correct this bias, we developed a Python script (Supporting Information, File S1) that called a heterozygote if both verified alleles had a depth of more than two and the total read depth at the locus was 10X or greater; this was the minimum depth we designated previously. Parental haplotypes for loci following Mendelian inheritance in the diploid cross were determined using linkage relationships with the previously mapped microsatellite markers.

Finally, we used 5′-nuclease genotyping as in Seeb *et al.* (2011) to screen and map 384 SNPs that originated from other labs (Smith *et al.* 2005a, b; Campbell and Narum 2008; Abadia-Cardoso *et al.* 2011; Larson *et al.* 2014) in two haploid families. Many of these loci are polymorphic expressed sequence tags (ESTs) that are used in conservation and management applications for Chinook salmon across Pacific North America (*e.g.*, Smith *et al.* 2005c; Hess *et al.* 2011; Templin *et al.* 2011; Matala *et al.* 2012).

#### Linkage group construction and alignment with Chinook chromosomes:

We used Onemap 2.0-3 (Margarido *et al.* 2007) for genome mapping in the haploid crosses and the F2 diploid cross. The Chinook salmon karyotype comprises 34 pairs of chromosomes (Phillips and Rab 2001). We therefore predicted 34 linkage groups per mapping cross. Linkage groups were identified independently for each haploid and diploid family using Onemap with a maximum recombination fraction of 0.25 and a starting LOD of 3.0. This LOD was subsequently increased by increments of 1.0 until the number of linkage groups identified was 34 or greater. We then used the microsatellite markers previously mapped and the RAD loci polymorphic in the diploid cross and the haploid crosses to identify each chromosome. Markers on each linkage group were subsequently ordered using Onemap for each haploid family. Individual haploid maps were merged using MergeMap (Wu *et al.* 2011) to create a consensus map.

#### Centromere mapping:

We estimated the proportion of heterozygous progeny in each gynogenetic diploid family at every non-duplicated marker mapped on the haploid map and polymorphic in the gynogenetic diploid crosses. This information was used to identify the centromere and the chromosome type (acrocentric or metacentric) for each haploid family. Comparison with the diploid map was used to characterize the short (p) arm and long (q) arm for each chromosome as defined in Phillips *et al.* (2013).

### Analysis of the properties of the Chinook salmon linkage groups

#### Frequency of recombination:

Recombination is usually reduced around the centromere in most species (Nachman 2002) and in the telomeric regions in the female in salmonids (Lien *et al.* 2011). Reduced recombination will result in a high number of loci mapping to the same position. Here, we examined the distribution of the markers along the linkage groups relative to the center of the centromere to determine recombination frequency.

#### Crossover frequency and interference:

Salmonids are thought to exhibit complete to near-complete interference (Thorgaard *et al.* 1983). We estimated the number of crossover events per chromosome arm using Linkmfex 2.3 (Danzmann 2005). Metacentric linkage groups were divided in two chromosome arms. For each chromosome arm we counted the number of progeny with 0, 1, or more crossovers. Absence of double crossovers on all chromosome arms for every progeny would confirm the hypothesis of complete interference.

#### Distribution of duplicated markers across the genome:

Two types of duplicated markers were used in this study. Duplicated loci with both paralogs polymorphic (BPP) were used to infer homeologies, because both paralogs could be mapped (Table 1). Occasional homeologous chromosome pairing in salmon may result in reduced divergence between the arms involved. We examined the position of the duplicated loci on the consensus haploid map to determine whether there was a bias in distribution of these loci. We reasoned that this analysis would identify chromosomal regions of reduced divergence between homeologs, indicating possible map positions where homeologs have a tendency to pair. Here, we estimated the relative proportion of duplicated loci along the linkage groups. Because map positions are not uniformly distributed along the chromosomes, we used a kernel smoothing sliding window approach with a bandwidth of 2cM to determine the relative proportion of duplicated loci along the linkage groups.

### Comparative mapping with rainbow trout

To examine differences in chromosomal arrangement between Chinook salmon and rainbow trout, we aligned the 40,649 RAD loci identified in the latter species (Miller *et al.* 2012) with the reference dataset of loci created for Chinook salmon. To achieve this goal, we used bowtie, allowing a maximum of three nucleotide mismatches per locus. Mapped loci in common between the two species were used to identify homologies between rainbow trout and Chinook salmon and confirm alignment with previous studies (Naish *et al.* 2013; Phillips *et al.* 2013).

## Results

### Reference database of RAD loci for Chinook salmon

A total of 62,249 putative loci were sequenced in at least 135 individuals from the Columbia River with a minimum depth of five reads per locus per individual: these sequences formed the temporary database of RAD loci. Of these, 2713 were removed because they did not align uniquely to themselves. After conducting a Blast search of the temporary database against itself, 1451 loci did not have a BLAST result or the best hit was not itself, mostly due to the presence of repetitive units in the sequence (data not shown). Alignments of all reads for the haploid individuals against the updated temporary database were not unique for 3148 loci and these were therefore removed from the database. Finally, 6409 duplicated loci were identified as heterozygous in more than two progeny in at least one haploid family and were identified as such. The final reference database comprised 48,528 putative non-duplicated loci and 6409 duplicated loci (File S2).

### Linkage mapping

#### Haploid and diploid linkage maps:

The three haploid families (here, family A, B, and C) had 3528, 3325, and 3403 biallelic polymorphic RAD loci respectively, representing 7146 unique RAD loci. Two families were genotyped using the 384 5′-nuclease panel (family A and B); each had 98 and 92 polymorphic SNPs respectively, 153 of which were unique. We used 2674 informative biallelic RAD loci scored in the diploid cross to develop sex-specific linkage groups. A subset of 1189 loci was polymorphic in the female parent and linked to previously mapped microsatellite markers (Naish *et al.* 2013). We identified 34 linkage groups corresponding to the chromosomes for each haploid cross using 578 RAD loci that were in common between the diploid and haploid families.

**Figure 1:**
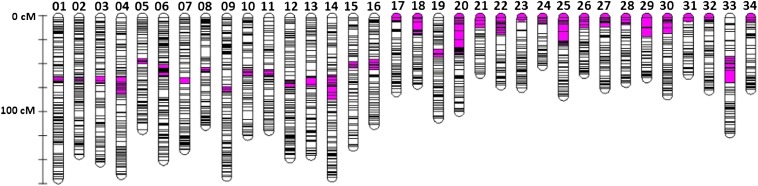
Consensus Chinook salmon female linkage map—34 linkage groups corresponding to the 34 Chinook salmon chromosomes. Ots01 to Ots16 are metacentric; Ots17 to Ots34 are acrocentric. The size of each linkage group varies from 58 to 173.2 cM. Each line corresponds to the location of one or more markers. The centromere is represented in pink. All the chromosomes are oriented with the shorter arm (p arm) before the centromere, longer arm (q arm) after the centromere.

We mapped 3485, 3291, and 3273 non-duplicated and duplicated markers within each of the three haploid families (Table 2). The map lengths ranged between 2834.9 cM and 3099.6 cM (Table 2). A total of 2319 loci were polymorphic in more than one family and were used to merge the haploid maps. The consensus haploid map comprised 7304 markers and measured 4163.9 cM (Figure 1 and File S3).

**Table 2:** Number of loci mapped and map length for each haploid family and for the consensus map

	Non-duplicated RAD Loci	OPP	BPP	SNP	Total	Map Length, cM
Family A	3001	324	62	98	3485	2834.9
Family B	2922	245	32	92	3291	3099.6
Family C	3011	230	32	−	3273	2991.8
Consensus map	6352	603	196	153	7304	4163.9

Four types of markers were used: non-duplicated RAD loci, duplicated RAD loci for which only one paralog was polymorphic (OPP) or both paralogs were polymorphic (BPP), and SNP loci. RAD, restriction-site associated DNA; SNP, single-nucleotide polymorphism.

The diploid map comprised 1101 non-duplicated RAD markers and 242 microsatellite loci (File S3). All 34 chromosomes were identified, but five chromosomes were represented by 2 linkage groups each (Ots08, Ots15, Ots19, Ots26, and Ots29). The number of individuals scored per locus was variable due to lower DNA quality. As a result, the marker order on the female diploid map was not consistently reliable. Therefore, the diploid map was not merged with the haploid maps. However, the microsatellite markers proved reliable in assigning linkage group arms to chromosomes.

#### Centromere mapping:

**Figure 2:**
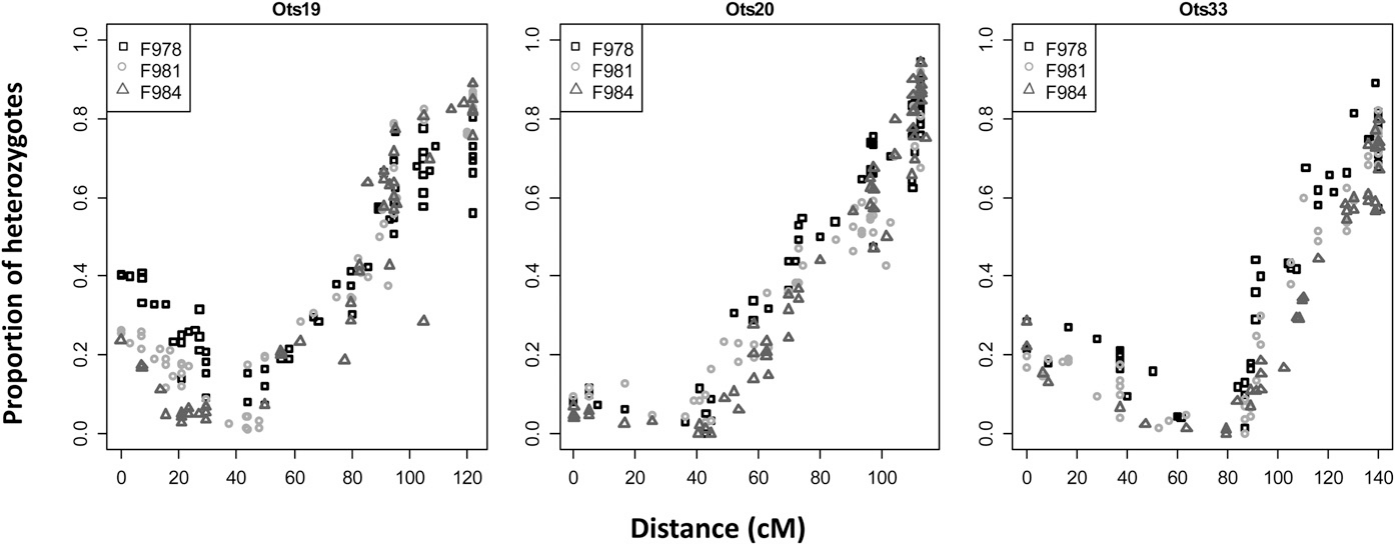
Percentage of heterozygous offspring in the gynogenetic diploid crosses along three acrocentric chromosomes where the p arm has been identified: Ots19, Ots20, and Ots33. On the x-axis, the distances are oriented from the p arm. Three gynogenetic crosses were used (F978, F981, and F984). The centromere is located where the percentage of heterozygous offspring is about zero.

Of the 6348 non-duplicated RAD markers placed on the haploid map, 3021 were polymorphic in at least one of the three gynogenetic diploid crosses and were used to identify the centromeres. Placement of the centromere permitted identification of 16 metacentric linkage groups (Ots01 - Ots16) and 18 acrocentric linkage groups (Ots17 to Ots34), corresponding to the known Chinook salmon karyotype (Figure 1 and File S4). The small (p) chromosome arm of acrocentric chromosomes (Phillips and Rab 2001) is usually uncharacterized in mapping studies because there are often insufficient markers describing this region. In this study, we identified the small arm for three acrocentric chromosomes (Ots19, Ots20, and Ots33; Figure 2). It is interesting to note that the linkage map sizes did not correlate with the sizes of the chromosomes, but the metacentric linkage groups (Ots01 to Ots16) were longer than the acrocentric linkage groups (Ots17 to Ots34). Ots19, Ots20, and Ots33 were the longest acrocentric linkage groups.

### Analysis of the properties of the Chinook salmon linkage groups

#### Frequency of recombination:

**Figure 3:**
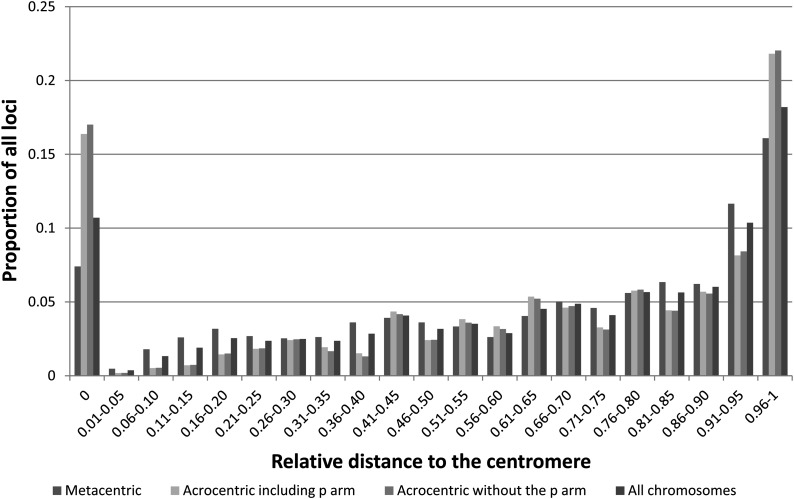
Marker distribution across all chromosome arms, examined separately for all chromosomes, metacentric chromosomes, and acrocentric chromosomes (including the p arm or not for Ots19, Ots20, and Ots 33). Distances on the x-axis are represented as the relative distance from the center of the centromeric region.

The distribution of markers across all chromosomes (Figure 3) revealed a bias in marker placement. The greatest numbers of mapped loci were placed at the centromeres and toward the telomeres; the number of markers increased with increasing distance from the centromere regardless of the type of chromosome (Figure 3). This over-representation of markers at distal positions suggests that there is reduced recombination in the telomeres relative to the remaining chromosomal regions in the female.

#### Crossover frequency and interference:

We used one haploid family (Family A) with 46 progeny to examine the number of crossovers in 50 chromosome arms (2300 chromosome arms). We only observed 60 instances (2.6%) of double crossovers. The occurrences of double crossovers were not randomly distributed between chromosomes. The chromosomes with the highest frequency of double crossovers were acrocentric. Double crossovers occurred in Ots19, Ots20, and Ots33, for 10, 6, and 6 progeny respectively. However, the second crossover always occurred on the short arm of these three chromosomes. The remaining double crossovers occurred on 21 chromosome arms (File S5). Finally, the frequency of double crossovers was not correlated to the number of duplicated loci on the linkage groups (*t*-test *P*-value: 0.42).

In gynogenetic diploid progeny, the maximum proportion of heterozygotes (MPH) at a locus in the telomeric region should be 0.67 if there is no interference and 1.00 if there is complete interference (Thorgaard *et al.* 1983). Here the average MPH for each chromosome arm was 0.90. The MPH ranged from 0.75 to 0.99, except for Ots11p, where the MPH was 0.49. Here, it was only possible to genotype the non-duplicated loci in the gynogenetic diploids. Given that the distal regions from the centromere of 16 chromosome arms mainly comprised duplicated loci, we did not have full coverage of these arms in the gynogenetic diploid crosses. Indeed, we observed that all the arms with the lowest MPH (<0.85) had a greater proportion of duplicated loci. Therefore, we concluded that the lower MPH observed for those arms was due to a lack of coverage with the gynogenetic diploid rather than absence of interference.

#### Duplicated loci and homeologies:

**Figure 4:**
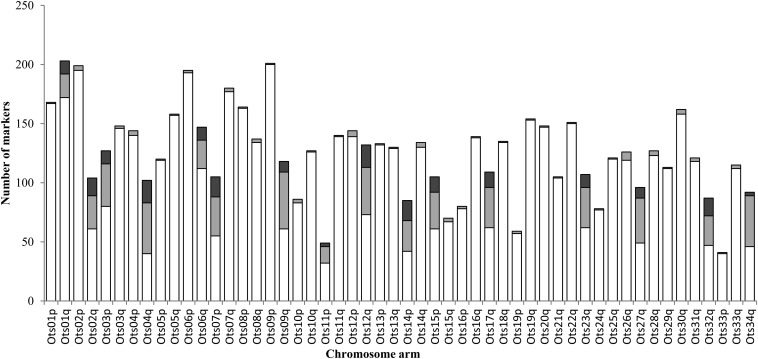
Number of markers on each linkage group, designated by chromosome arm. Non-duplicated loci (RAD loci or SNP loci) are represented by the white bars; Duplicated loci are represented by the light gray bars (loci with only one paralog polymorphic) or dark gray bars (both paralogs polymorphic).

**Figure 5:**
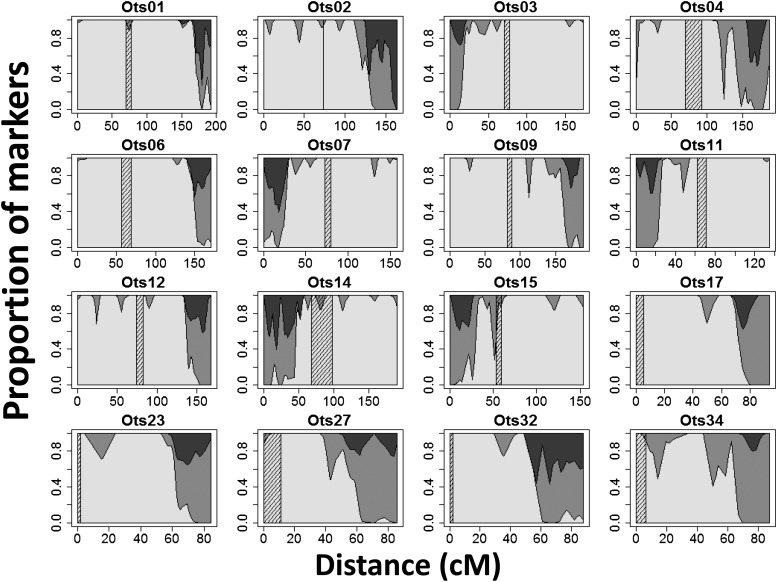
Proportion of duplicated and non-duplicated loci along the 16 linkage groups (denoted by chromosome number) with a high number of duplicated loci. Non-duplicated loci are represented in white; Duplicated loci are represented in gray (loci with one paralog polymorphic) or in dark gray (loci with both paralogs polymorphic). The centromere is represented by the cross-hatched area. All chromosomes are oriented with the short arm (p) on the left where relevant.

A total of 799 duplicated loci detected by RAD sequencing were placed on the linkage map. The duplicated loci were not distributed uniformly between the chromosomes. We observed two categories of chromosome arms: those with very few duplicated loci (1−7 loci, corresponding to 0.5–5.6% of all duplicated markers in the data) and those with many duplicated loci (17−62 loci, corresponding to 15–61% of all markers). A total of 89.7% of the duplicated loci were located on 16 chromosome arms (Figure 4). Homeologies were inferred between eight pairs of chromosome arms using 98 paralogs that were polymorphic at both loci (Table 3). Six of the homeologies had been identified by Naish *et al.* (2013), but two were novel (Ots01q/06q and Ots07p/14p). Ots01q and Ots06q had the lowest number of duplicated markers: 15% and 24% of the markers mapping to these linkage groups respectively were duplicated. All other chromosome arms had between 35% and 61% loci that were duplicated. Finally, the duplicated loci were not evenly distributed along the 16 chromosome arms that had a greater number of these loci. The regions distal from the centromere almost exclusively comprised duplicated loci (Figure 5).

**Table 3:** Homeologous chromosome pairs identified for Chinook salmon, Atlantic salmon, or both

Chinook Salmon Homeologs	Chinook Salmon Linkage Groups	Number of Marker Pairs Supporting Homeolog Pairings	Homeology in Atlantic Salmon	Number of Markers and Type of Support for the Homeology in Lien *et al.* (2011)	Rainbow Trout Homeologs
1. High numbers of duplicated markers in Chinook and Atlantic salmon					
Ots03p-Ots23	Ck05-Ck25	11	Ssa02p-Ssa05q	39 MSV5	Omy03p-Omy02p
Ots15p-Ots17	Ck23-Ck01	13	Ssa07q-Ssa17qb	33 MSV5	Omy21p-Omy15q
Ots09q-Ots27	Ck02-Ck31	9	Ssa03q-Ssa06q	7 MSV5	Omy12q-Omy13q
Ots11p-Ots34	Ck15-Ck32	3	Ssa04p-Ssa08q	14 MSV5	Omy19p-Omy10q
2. Higher numbers of duplicated markers in Chinook salmon compared with Atlantic salmon					
Ots01q-Ots06q	Ck13-Ck17	11	Ssa01qa-Ssa18qa	BLAST	Omy23-Omy01q
Ots02q-Ots32	Ck12-Ck30	15	Ssa02q-Ssa12qa	1 MSV5	Omy17p-Omy13p
Ots04q-Ots12q	Ck08-Ck18	19	Ssa26-Ssa11qa*^a^*	2 MSV5	Omy06q-Omy26
Ots07p-Ots14p	Ck16-Ck10	17	Ssa17qa-Ssa16qb*^a^*	BLAST	Omy07p-Omy18p
3. Homeologies not observed in Chinook salmon and supported by only one duplicated marker in Atlantic salmon					
Ots22-Ots16q*^a^*	Ck34-Ck04	0	Ssa13qa-Ssa15qb*^a^*	1 MSV5	Omy16q-Omy09q
Ots24-Ots29*^a^*	Ck27-Ck03	0	Ssa19qb-Ssa29*^a^*	1 MSV5	Omy16p-Omy15p

Chinook salmon linkage groups, number of pairs of markers supporting the homeologies, corresponding homeologs in Atlantic salmon and type of support for the homeologies in Lien *et al.* (2011), and corresponding homeologies in rainbow trout are represented. Support for the homeologies in Lien *et al.* (2011): duplicated SNP loci with both paralog polymorphic (MSV5) or alignment-based using BLAST within Atlantic salmon or with stickleback. Note: we have corrected the homeology between Omy15q and Omy21p that was incorrectly reported as being between Omy15q and Omy21q in Phillips *et al.* (2006) and in subsequent studies (Phillips, personal communication). SNP, single-nucleotide polymorphism.

a Homeology between two acrocentric chromosomes.

### Comparison with rainbow trout

**Figure 6:**
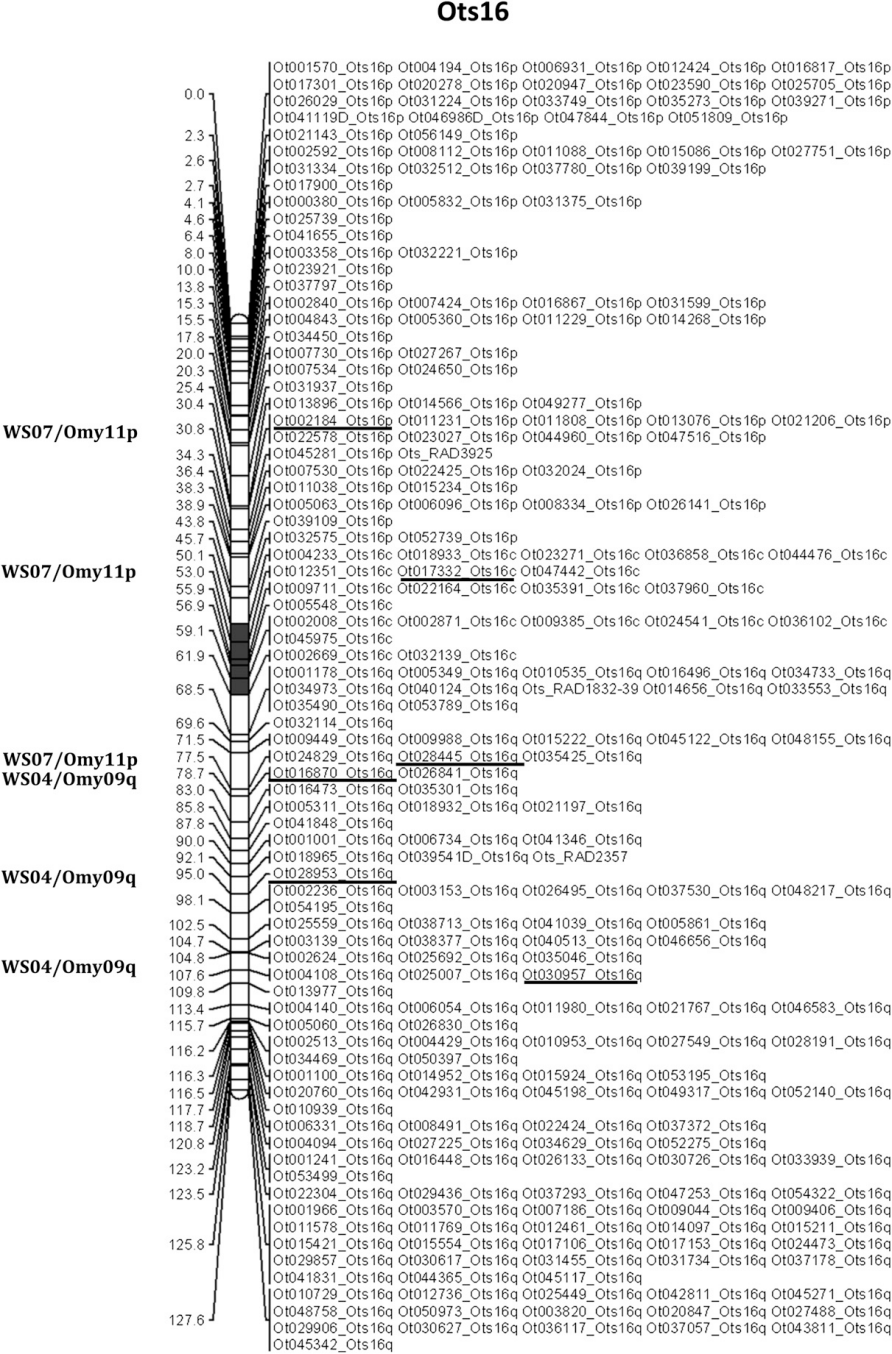
Linkage map for Ots16, denoting loci that are homologous with rainbow trout. Loci in common between the two species are underlined and the position on the rainbow trout map is indicated on the left of the chromosome (WS: linkage groups from Miller *et al.* 2012; Omy: rainbow trout chromosomes). The centromere is represented in gray. The chromosome is oriented with the p arm on top and the q arm at the bottom. Distances are in centimorgans.

A total of 40,649 RAD loci have been described in rainbow trout (Miller *et al.* 2012). More than 50% of these loci (20,436) aligned uniquely to the non-duplicated markers in the Chinook salmon reference database. A total of 317 RAD loci mapped in both species, allowing us to confirm previously described homologies between the two species (Naish *et al.* 2013) (Table 4). We confirmed the speculation in Naish *et al.* (2013) that Ck04 (Ots16) is homologous to rainbow trout linkage groups Omy11p and Omy09q, a finding also in agreement with the observations in Phillips *et al.* (2013). These earlier studies showed that Ots16p and Ots16q are homologous to a portion of Omy11p and Omy9q respectively. Here we observed one marker from Omy11 on Ots16q (Figure 6). We were not able to compare the order of the RAD loci between the rainbow trout map and the Chinook salmon map because most of the markers polymorphic in both species on a linkage group mapped to a single position on the rainbow trout map, which is based on a androgenetic doubled haploid cross.

**Table 4:** Homologies between Chinook salmon and rainbow trout chromosome arms and number of RAD markers supporting the homologies in this study

Chinook chromosome (current study, Phillips *et al.* 2013)	Chinook linkage group (Naish *et al.* 2013)	Rainbow trout Chromosome (Phillips *et al.* 2006)	Rainbow trout linkage group (Miller *et al.* 2012)	Number of markers supporting homology
Ots01p	Ck13	Omy04p	WS01	7
Ots01q		Omy23	WS27	6
Ots02p,q	Ck12	Omy17p,q	WS23	13
Ots03p,q	Ck05	Omy03p,q	WS06	7
Ots04p,q	Ck08	Omy06p,q	WS13	15
Ots05p	Ck11	Omy08p	WS05	5
Ots05q		Omy05q	WS03	18
Ots06p,q	Ck17	Omy01p,q	WS20	17
Ots07p,q	Ck16	Omy07p,q	WS25	5
Ots08p,q	Ck14	Omy25	WS24	19
Ots09p,q	Ck02	Omy12p,q	WS21	13
Ots10p	Ck20	Omy09p	WS04	2
Ots10q		Omy08q	WS05	8
Ots11p,q	Ck15	Omy19p,q	WS22	7
Ots12p	Ck18	Omy11p+q	WS07	8
Ots12q		Omy26	WS28	2
Ots13p	Ck07	Omy18q	WS19	4
Ots13q		Omy27	WS27	8
Ots14p	Ck10	Omy18p	Ø	0
Ots14q		Omy24	WS17	11
Ots15p,q	Ck23	Omy21p,q	WS26	9
Ots16p	Ck04	Omy11p	WS07	3
Ots16q		Omy09q	WS04	3
Ots17	Ck01	Omy15q	WS12	8
Ots18	Ck33	Omy04q	WS01	6
Ots19	Ck22	Omy02q	WS18	12
Ots20	Ck28	Omy05p	WS03	11
Ots21	Ck09	Omy14q	WS10	6
Ots22	Ck34	Omy16q	WS08	6
Ots23	Ck25	Omy02p	WS18	2
Ots24	Ck27	Omy16p	WS08	8
Ots25	Ck06	Omy20p+q	WS15	6
Ots26	Ck21	Omy22	WS02	9
Ots27	Ck31	Omy13q	WS29	2
Ots28	Ck24	Omy28	WS09	9
Ots29	Ck03	Omy15p	WS12	5
Ots30	Ck29	Omy10p	WS14	11
Ots31	Ck26	Omy14p	WS10	6
Ots32	Ck30	Omy13p	WS29	4
Ots33	Ck19	OmySex	WS11	7
Ots34	Ck32	Omy10q	WS14	3

RAD, restriction-site associated DNA.

## Discussion

The aim of this study was to characterize the evolution of Chinook salmon chromosomes relative to that of other salmonids following a whole duplication event in a common ancestor. This goal was achieved by improving the genomic resources for the species and by performing comparative mapping. We have developed a reference database of RAD loci for Chinook salmon comprising 48,528 non-duplicated loci and 6409 known duplicated loci. We identified 7151 polymorphic RAD loci in three haploid families that were used, along with 153 SNP loci currently used in conservation and management studies, to create a consensus map with a length of 4163 cM. The map comprised 34 linkage groups, which were anchored to all Chinook salmon chromosome arms using microsatellite loci that have been physically mapped in previous studies. The placement of 799 duplicated loci on the linkage map revealed an uneven distribution of these loci across chromosomes, suggesting that homeologs diverged at different rates following whole genome duplication. Crossover frequency measured in one haploid family confirmed near complete interference across chromosome arms. Finally, the genome map supports previously published homologies among rainbow trout and Chinook salmon chromosome arms, but these homologies are supported using more extensive data and centromere placement.

The reference database of RAD loci in Chinook salmon is extensive and provides a resource against which future RAD sequences generated using *Sbf*I as a restriction enzyme can be aligned. Markers that were polymorphic in the mapping families have been annotated in the database by chromosome arm. We attempted as far as possible to identify loci that had repeat units or were located in repeat regions using a series of screening tests based on self-alignment. However, the use of three haploid families would not have identified paralogs in the database that were not polymorphic. Therefore, we recommend aligning initial sequences generated in future studies to the reference database, and treating a locus that aligns to more than one of the reference loci as a putative duplicate.

The coverage of the Chinook salmon linkage map, 7304 markers that span all 34 chromosomes, is comparable to published maps in other salmonids. For example, the map for Atlantic salmon (*Salmo salar*) comprises 5650 SNPs (Lien *et al.* 2011), for rainbow trout 4563 RAD markers (Miller *et al.* 2012) and for sockeye salmon 1672 RAD markers (Everett *et al.* 2012). The present map had a size of 4163.9 cM, which is significantly larger than the first generation map available for Chinook salmon (2206.2 cM for the sex average map; Naish *et al.* 2013). The distances differ from the most recent maps for Atlantic salmon (2402.3 cM for females and 1746.2 cm for males; Lien *et al.* 2011) and rainbow trout (3600 cM; Guyomard *et al.* 2012). There are three reasons that might explain the differences. Nonrandom missing values (Jorgenson *et al.* 2005) and genotyping errors (Hackett and Broadfoot 2003) can inflate map distances. We found that missing values were not randomly distributed across individuals (χ^2^ test for uniform distribution across individuals: P-value ~0 for each family). We also noted earlier that genotyping RAD markers may be biased against heterozygotes. As a result, the genotyping error for the duplicated loci can be predicted to be higher for such loci. Finally, the extensive addition of duplicated markers at the telomeres might have increased map length. On the other hand, we also acknowledge that relatively few individuals were mapped (four families with 44−72 offspring). While this study demonstrates the possibility of constructing dense and high-resolution maps with relatively small sample sizes, increasing the number of individuals per family and number of families will result in better mapping resolution, with fewer loci mapping to the same position. While the Atlantic salmon map of Lien *et al.* (2011) comprised 3297 fish from 143 families, our sample sizes are comparable to other high-density salmon maps [*e.g.*, rainbow trout, two families of 60 individuals (Guyomard *et al.* 2012) and one family of 123 individuals (Miller *et al.* (2012); sockeye salmon, one family of 96 individuals to create the initial RAD-EST linkage map, and an additional 13 families with 45 or 93 individuals to increase the number of EST loci (Everett *et al.* (2012)].

We located the centromere for 18 acrocentric chromosomes and 16 metacentric chromosomes, using more than 3000 loci for three gynogenetic diploid families. The centromeric regions were sometimes large. The percentage of heterozygote offspring was constrained by the number of progeny in each cross (84−93). Increasing the number of crosses, as well as the number of progeny, would facilitate the narrower placement of the centromere relative to the mapped markers. The location of the centromere allowed us to conclusively support previous findings on chromosome arm arrangement in Chinook salmon (Phillips and Rab 2001; Phillips *et al.* 2013). Additionally, we confirmed that Ots25 (Chinook salmon linkage group Ck06) was acrocentric and that Ots16 (Ck04) was metacentric, as speculated in Naish *et al.* (2013) and Phillips *et al.* (2013). Six of the homeologous chromosome pairs detected in this study had been previously identified in Chinook salmon, two were novel (Ots01q/06q and Ots07p/14p) and highly supported, and three previously identified pairings were not observed here (Table 3). Eleven homeologous chromosome arm pairs have therefore been identified to date for Chinook salmon.

Our data support the hypothesis of near complete interference in Chinook salmon, where we observed very few occurrences of double crossovers and a maximum proportion of heterozygotes close to one for all chromosomes in the gynogenetic diploid families. This result agrees with previous studies (*e.g.*, Guyomard *et al.* 2006) but is supported by a much higher number of markers and recombination events observed. We also observed that the frequency of recombination was reduced in the telomeric regions in females, as suggested in Moen *et al.* (2004), Danzmann *et al.* (2008), or Lien *et al.* (2011). The greater proportion of markers mapping in the telomeric regions suggest that the map created in this study covers the entire genome, but that the order of the markers in the telomeres is likely not fully resolved, but could be by mapping male meiosis. Indeed, the male-based map based on RAD markers in rainbow trout (Miller *et al.* 2012) showed that most recombination events occurred at the telomeres.

Placement of the centromeres on the Chinook salmon linkage map and comparisons with the rainbow trout linkage map (Miller *et al.* 2012) confirmed all rearrangements and homologies previously identified (Naish *et al.* 2013; Phillips *et al.* 2013). Our data also support the fact that Ots16 (Ck04) comprises a fusion between a fragment of one chromosome arm from a metacentric chromosome, Omy11p, and another, Omy9q. However, the greater resolution on the current map shows that markers from Omy11p are found on both arms of Ots16, suggesting that there may have been a centromeric inversion on Ots16. The number of RAD loci shared between Chinook salmon and rainbow trout suggests that determining chromosome evolution across salmonids is increasingly feasible as more species are mapped using RAD loci. Since chromosome arms are mainly conserved across species, this map can also be used for genome-wide studies in other salmon species.

In this study, we examined the chromosomal distribution of duplicated loci that differed only at one, two, or three nucleotide sites. Thus, we assumed that these loci had only recently diverged or were still involved in occasional multivalent pairings. The distribution of this type of duplicated locus varied across chromosomes. Linkage group arms had either almost no duplicated loci, or they had a high density of duplicated loci primarily located in distal regions from the centromere. Population genetic studies based on RAD loci in duplicated regions might therefore be limited, and so we recommend using mapped ESTs or microsatellites to target these regions. Interestingly, all of these pairings involved at least one metacentric chromosome. The results suggest that divergence rates of homeologs following WGD have not been uniform. Comparative mapping shows that the homeologous pairings we identified in Chinook salmon have also been shown in other salmonids (Sakamoto *et al.* 2000; Danzmann *et al.* 2005; Gharbi *et al.* 2006; Danzmann *et al.* 2008; Lien *et al.* 2011).

Although qualitative, it is possible to speculate whether the relative rates of divergence between homeologous chromosome arms are consistent between Chinook salmon and Atlantic salmon, by comparing this study to that of Lien *et al.* (2011). Both studies mapped loci where both paralogs were polymorphic (here, designated “BPP,” and in Atlantic salmon, “MSV5,” Table 3) and we can broadly use these loci as a metric for reduced divergence between homeologs. Four pairs of chromosome arms have a large number of polymorphic paralogs mapped in both species (Table 3). An additional four pairs were highly supported in Chinook salmon but had no or few equivalent polymorphic loci in Atlantic salmon (1 or 2 MSV5 or arms were instead confirmed as homeologous by a BLAST search between Atlantic salmon and stickleback *Gasterosteus aculeatus*). Finally, two homeologies in Atlantic salmon, each supported by only one MSV5, were not observed in Chinook salmon. Importantly, the evidence for reduced divergence or ongoing recombination in the remaining chromosome arms in both species is small. There are three possible explanations for these observations. The first is that the patterns observed may simply be explained by marker density—these reported differences might diminish with extensive sequencing. The second is methodological; the duplicated loci in Atlantic salmon were mapped using SNP markers with two alleles, whereas the present study mapped paralogs that had up to four alleles. Loci in Chinook salmon were considered duplicated if the paralogs had a maximum of three substitutions. Relaxing the alignment parameters, or using SNPs with more alleles in Atlantic salmon, might permit identification of duplicated loci that have comparable polymorphisms. The third explanation is intriguing; namely, that the differentiation between the majority of homeologous pairs and the retention of pairing in some pre-dates the divergence between *Salmo* and *Oncorhynchus*. May and Johnson (1990) observed conservation of homeologous pairing between the same chromosomes across different salmon species. Our interpretation supports these observations.

Several studies have shown that the other main process of diploidization—chromosomal arm rearrangement—is extensive subsequent to divergence between the salmonid genera (Danzmann *et al.* 2005; Phillips *et al.* 2009; Timusk *et al.* 2011). Some homeologous pairings may have been prevented by these rearrangements. Wright *et al.* (1983) observed that ongoing homeologous pairing in salmon occurred between one acrocentric and one metacentric chromosome. Our results support this observation and add that metacentric-metacentric pairing also occurs. The involvement of at least one metacentric might provide the stability required the formation of multivalents. In Atlantic salmon, the q arm of some chromosomes has been formed by the fusion of two acrocentric chromosomes. In these cases the distal arm (qb) might be involved in homeologous pairing, but the proximal arm (qa) cannot. In conclusion, it is unclear whether the differences in divergence rates among chromosomes can simply be explained by homeologous pairing or whether selection has acted differentially across the genome following the WGD event (Mayfield-Jones *et al.* 2013), but elucidating these mechanisms can be explored by explicitly testing for evidence of selection at the molecular level.

Here, we developed two major genomic resources for Chinook salmon: a reference database of RAD loci and a very dense linkage map anchored to the chromosomes, where arms have been identified by placement of the centromeres. We have also identified homeologous chromosomal arm regions that appear to be less diverged than other pairs, highlighting areas that may be of interest in evolutionary analyses of residual polyploidy. These resources will facilitate genome-wide studies in Chinook salmon, such as genome scans (*e.g.*, Tsumura *et al.* 2012; Bradbury *et al.* 2013), QTL mapping (*e.g.*, Collard *et al.* 2005; Nichols *et al.* 2008), and genome-wide association analyses (*e.g.*, Cichon *et al.* 2009; Magwire *et al.* 2012), as well as studies in related salmon species.

## Supplementary Material

Supporting Information
