# Supplementary material for: A Dense Linkage Map for Chinook salmon (Oncorhynchus tshawytscha) Reveals Variable Chromosomal Divergence After an Ancestral Whole Genome Duplication Event
Source: G3 (Bethesda). 2013 Dec 30;4(3):447–60. doi: 10.1534/g3.113.009316 (PMC3962484; doi:10.1534/g3.113.009316)
Supplement: Supporting Information [file supp_4_3_447__index.html]

A Dense Linkage Map for Chinook salmon (Oncorhynchus tshawytscha) Reveals Variable Chromosomal Divergence After an Ancestral Whole Genome Duplication Event — Supporting Information 

# A Dense Linkage Map for Chinook salmon (*Oncorhynchus tshawytscha*) Reveals Variable Chromosomal Divergence After an Ancestral Whole Genome Duplication Event

## Supporting Information for Brieuc *et al.*, 2014

**Files in this Data Supplement:**

- Supporting Information - Files S1-S5 (PDF, 904 KB)
- File S4 - Percentage of heterozygous offspring in the gynogenetic diploid crosses along all chromosomes. (PDF, 319 KB)
- File S1 - Script to verify the genotypes inferred with STACKS (.py, 5 KB)
- File S2 - Database of RAD loci for Chinook salmon (.xlsx, 2 MB)
- File S3 - Linkage maps and mapped loci (.xlsx, 658 KB)
- File S5 - Number of individuals from Family A (n = 46) with double crossovers (DCO) for each chromosome arm. (.xlsx, 9 KB)
