# Supplementary material for: A Dense Linkage Map for Chinook salmon (Oncorhynchus tshawytscha) Reveals Variable Chromosomal Divergence After an Ancestral Whole Genome Duplication Event
Source: G3 (Bethesda). 2013 Dec 30;4(3):447–60. doi: 10.1534/g3.113.009316 (PMC3962484; doi:10.1534/g3.113.009316)
Supplement: Supporting Information [file supp_g3.113.009316_009316SI.pdf]

G3·Genes | Genomes | Genetics

A dense linkage map for Chinook salmon (*Oncorhynchus tshawytscha*) reveals variable chromosomal divergence following an ancestral whole genome duplication event.

Marine S.O. Brieuc, Charles D. Waters, James E. Seeb, Kerry A. Naish

School of Aquatic and Fishery Sciences, University of Washington, Seattle, WA, 98195-5020

Corresponding Author: Marine S.0. Brieuc; School of Aquatic and Fishery Sciences, University of Washington, UW Box 355020, Seattle, WA, 98195-5020, USA; Phone: (206) 616-3148; mbrieuc@uw.edu

DOI: 10.1534/g3.113.009316

Files S1-S3

Available for download at http://www.g3journal.org/lookup/suppl/doi:10.1534/g3.113.009316/-/DC1

File S1 Script to verify the genotypes inferred with STACKS

File S2 Database of RAD loci for Chinook salmon

S2.1: Non-duplicated loci in the reference database and sequences. Mapped markers have the chromosome arm added to the name (OtXXXXXX_OtXX[p, c or q]). Markers mapped in the diploid cross only, but for which the arm was not identified have the chromosome preceded by I (inferred; OtXXXXXX_IOtsXX)

S2.2: Duplicated loci in the reference database and sequences. The letter D was added after the name. Mapped markers also have the chromosome arm(s) annotated

S2.3: Loci removed from the temporary reference database during screening and sequences.

File S3 Linkage maps and mapped loci

S3.1: Consensus haploid map

S3.2-S3.4: Haploid maps for Family A, B and C

S3.5: Diploid map

S3.6: Position and alleles of the mapped loci

File S4

Percentage of heterozygous offspring in the gynogenetic diploid crosses along all chromosomes. On the x axis the distances are oriented from the p arm. Three gynogenetic crosses were used (F978, F981 and F984). The centromere is located where the percentage of heterozygous offspring is about zero.

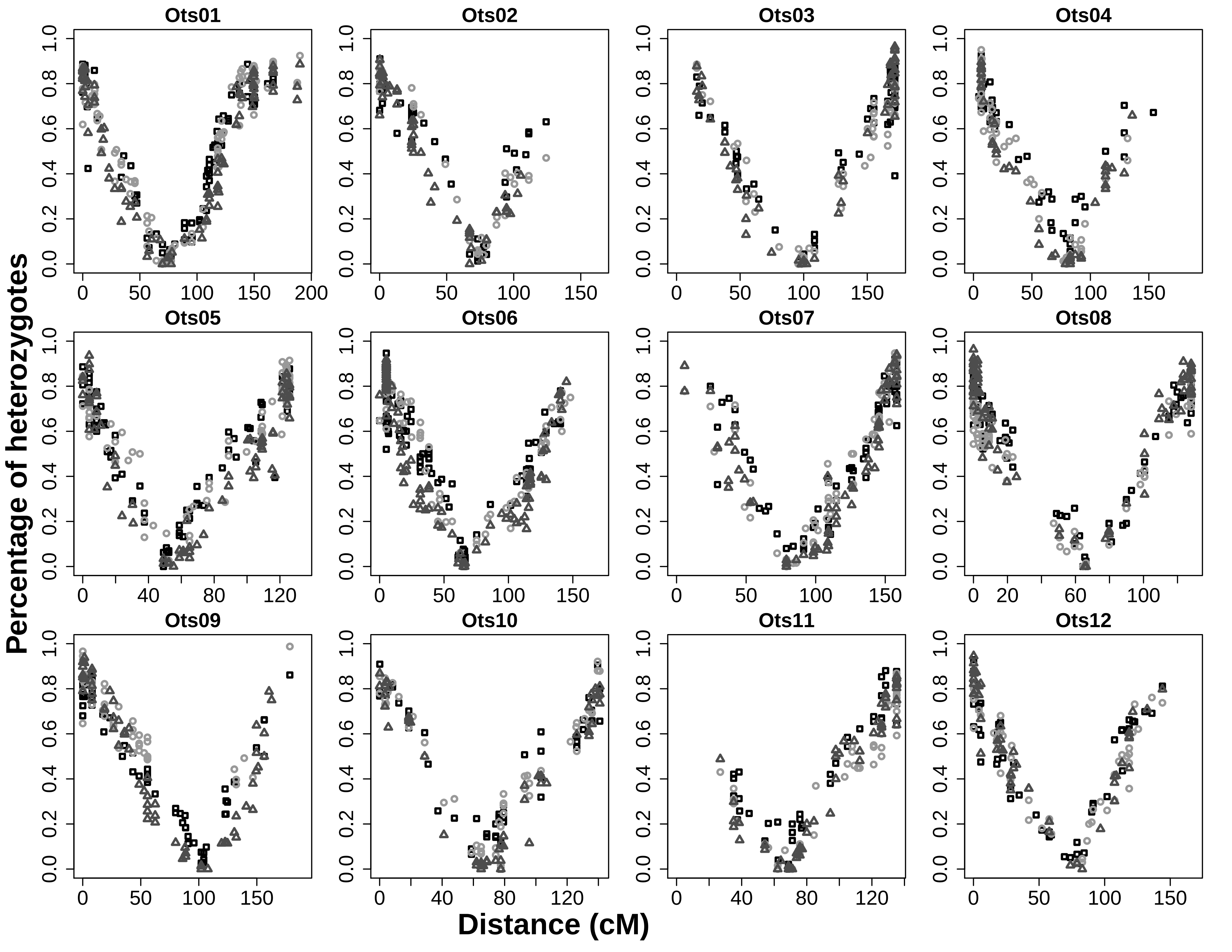

Ots01
Ots02
Ots03
Ots04
Ots05
Ots06
Ots07
Ots08
Ots09
Ots10
Ots11
Ots12
Percentage of heterozygotes
Distance (cM)

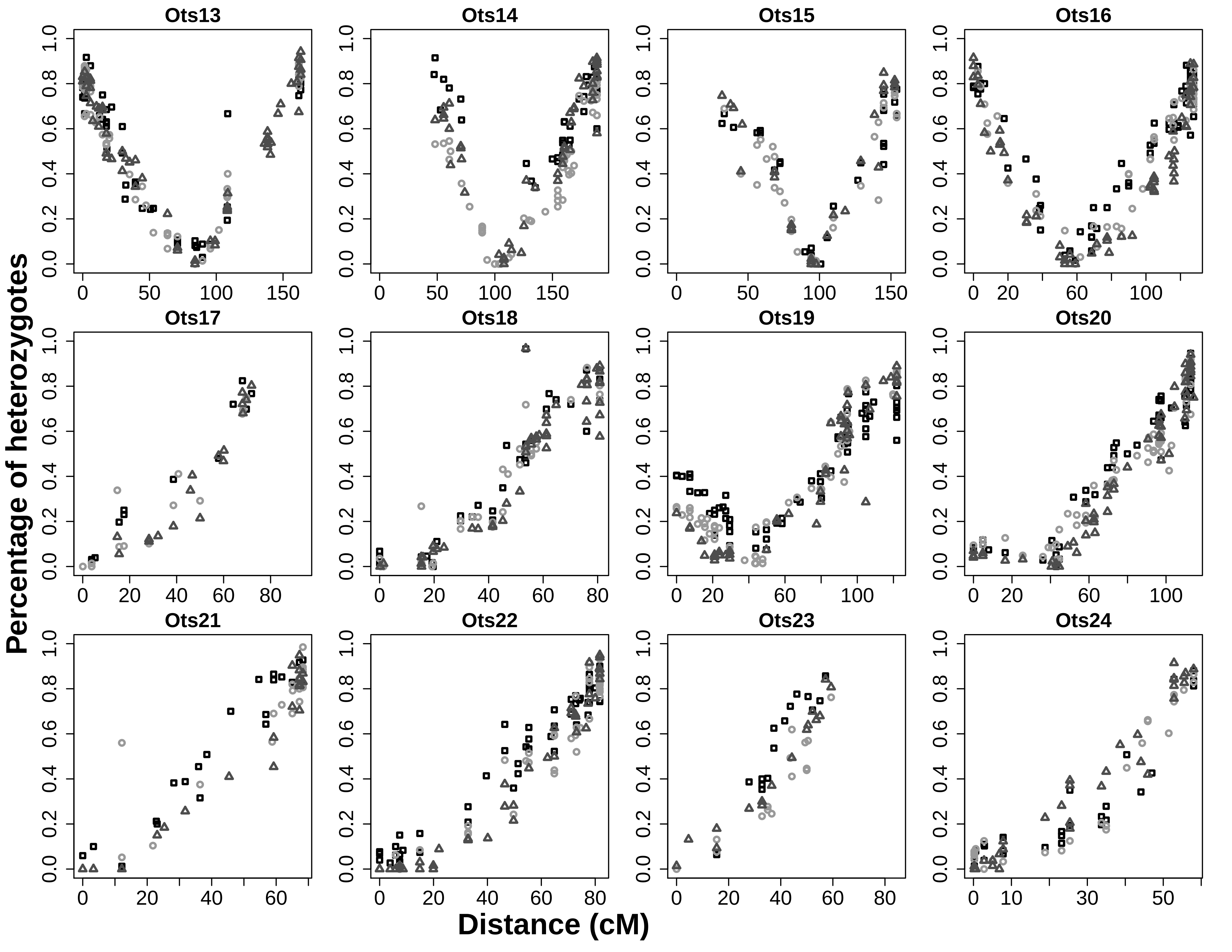
Ots13
Ots14
Ots15
Ots16
Ots17
Ots18
Ots19
Ots20
Ots21
Ots22
Ots23
Ots24
Percentage of heterozygotes
Distance (cM)

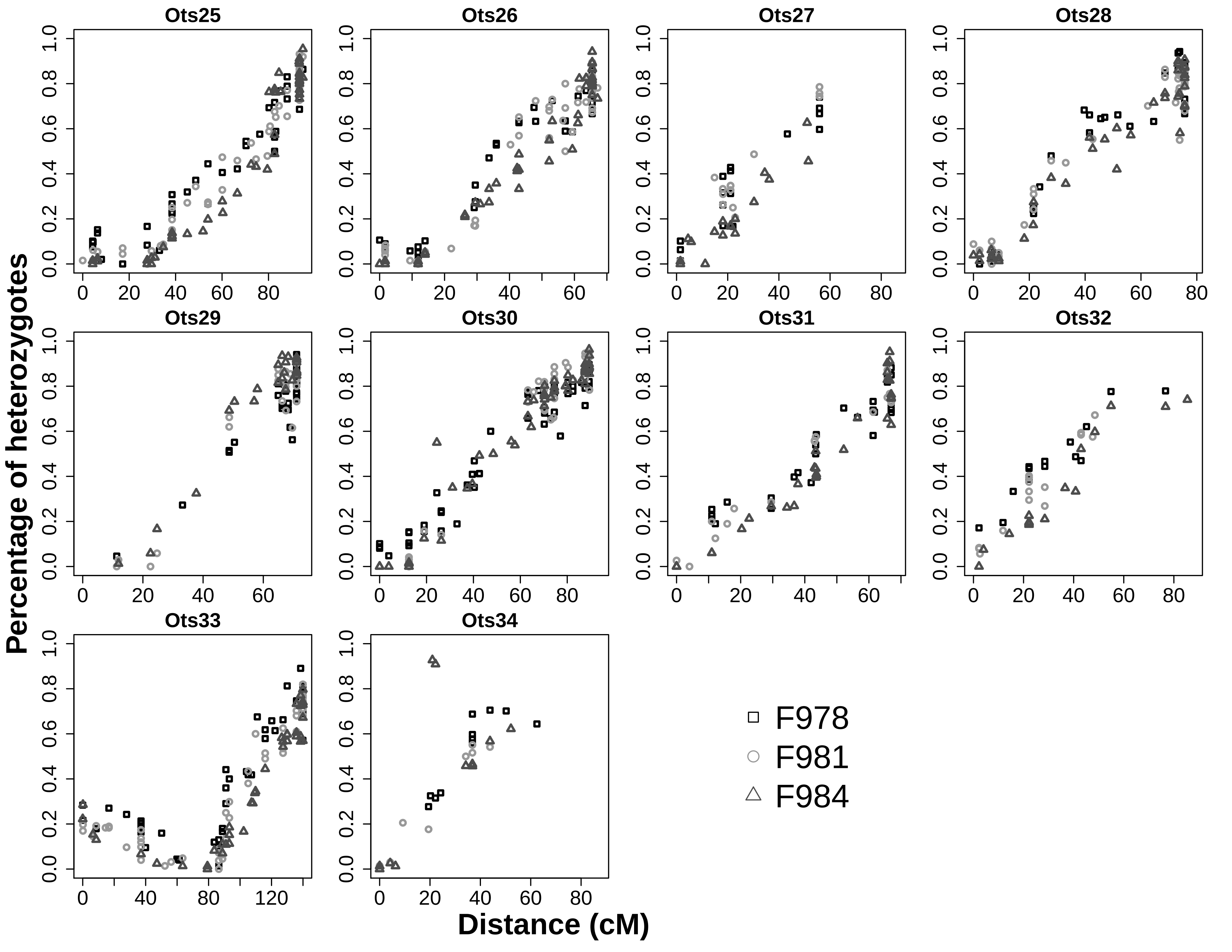
Ots25
Ots26
Ots27
Ots28
Ots29
Ots30
Ots31
Ots32
Ots33
Ots34
Percentage of heterozygotes
Distance (cM)
F978
F981
F984

File S5

Available for download as an Excel file at http://www.g3journal.org/lookup/suppl/doi:10.1534/g3.113.009316/-/DC1

Number of individuals from Family A (n = 46) with double crossovers (DCO) for each chromosome arm. *: A crossover occurred in the centromere of Ots12. It was not possible to attribute the crossover to one arm or the other and so the range of individuals with double crossovers for each arm was estimated.
