# Supplementary material for: A Dense Linkage Map for Chinook salmon (Oncorhynchus tshawytscha) Reveals Variable Chromosomal Divergence After an Ancestral Whole Genome Duplication Event
Source: G3 (Bethesda). 2013 Dec 30;4(3):447–60. doi: 10.1534/g3.113.009316 (PMC3962484; doi:10.1534/g3.113.009316)
Supplement: Supporting Information [file supp_g3.113.009316_FileS4.pdf]

File S4

Percentage of heterozygous offspring in the gynogenetic diploid crosses along all chromosomes. On the x axis the distances are oriented from the p arm. Three gynogenetic crosses were used (F978, F981 and F984). The centromere is located where the percentage of heterozygous offspring is about zero.

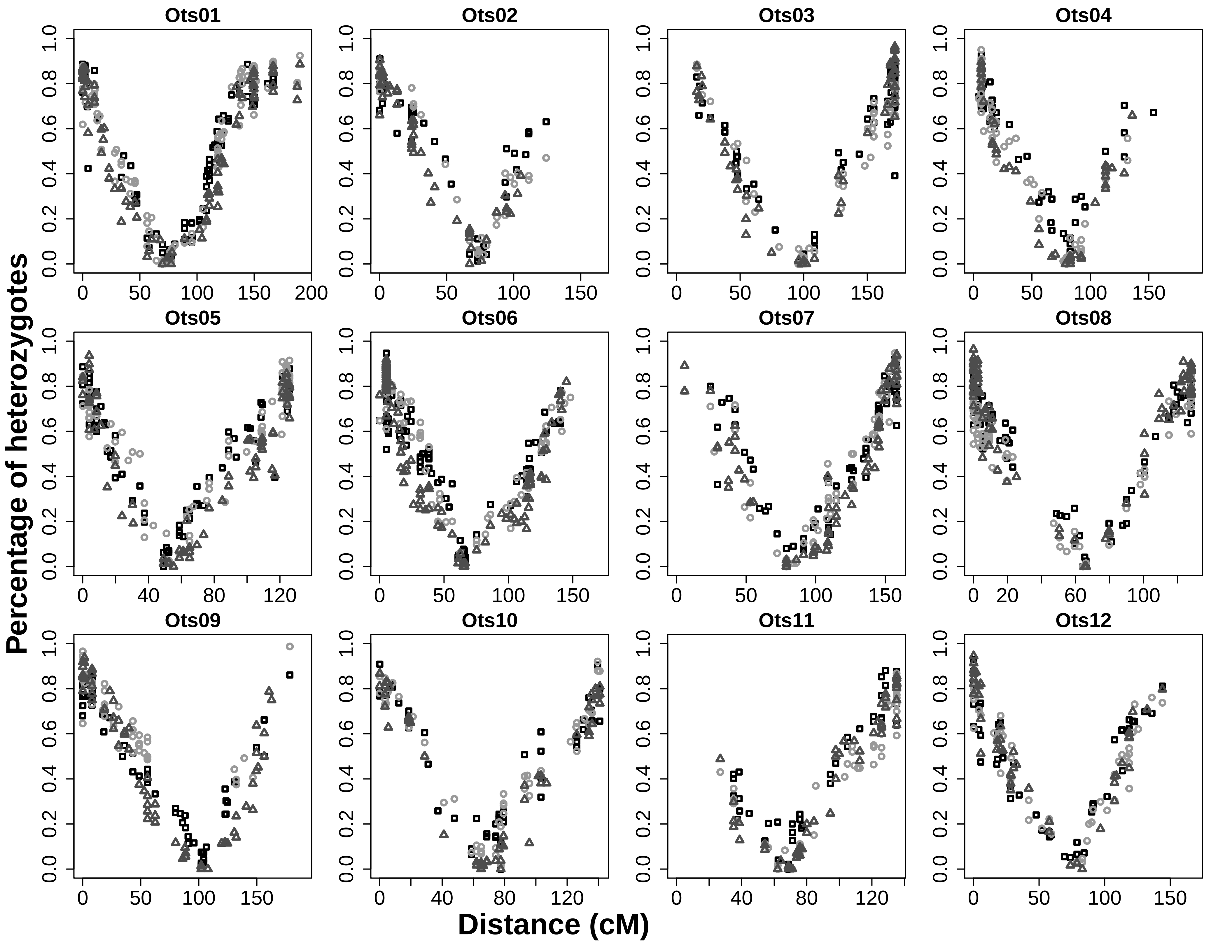
Ots01
Ots02
Ots03
Ots04
Ots05
Ots06
Ots07
Ots08
Ots09
Ots10
Ots11
Ots12
Percentage of heterozygotes
Distance (cM)

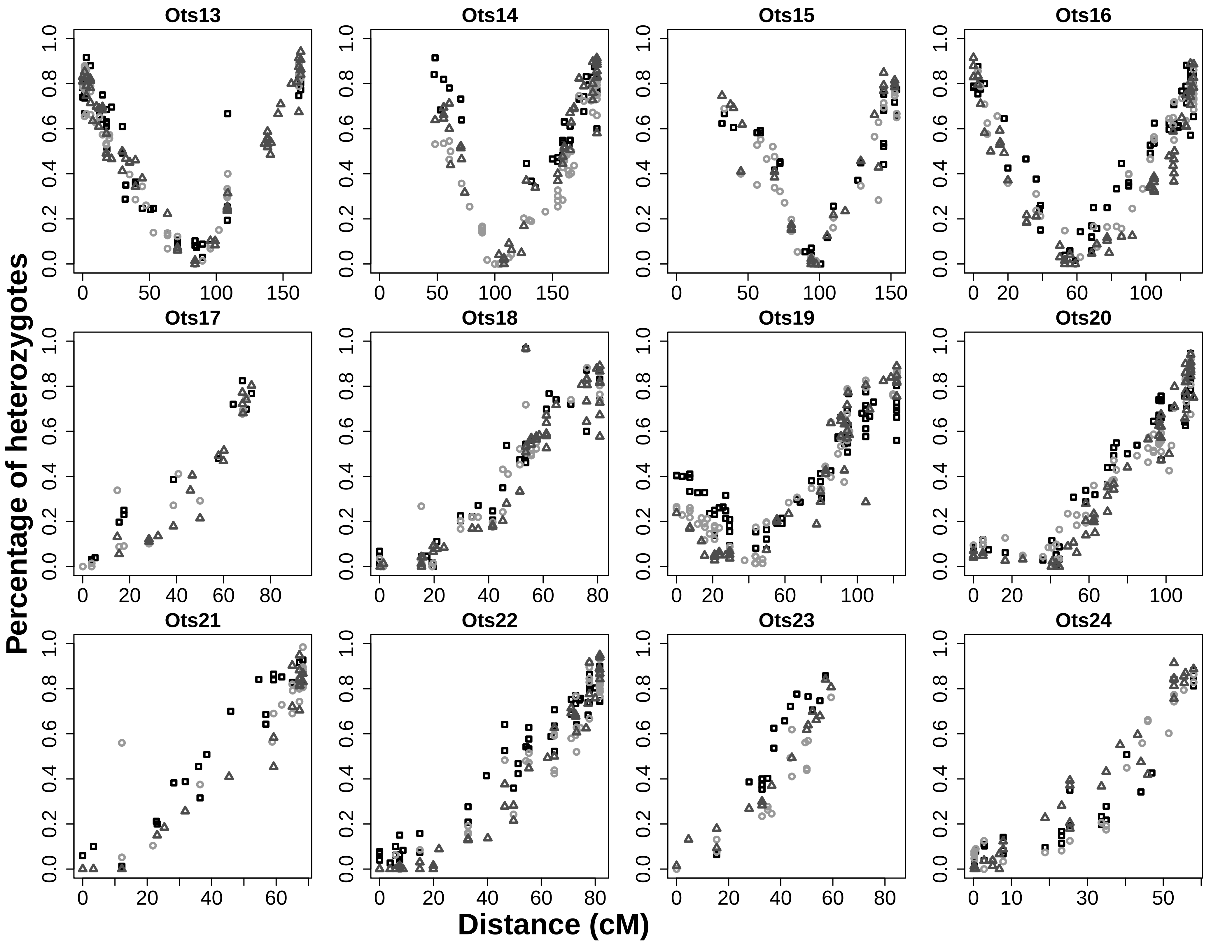

Ots13
Ots14
Ots15
Ots16
Ots17
Ots18
Ots19
Ots20
Ots21
Ots22
Ots23
Ots24
Percentage of heterozygotes
Distance (cM)

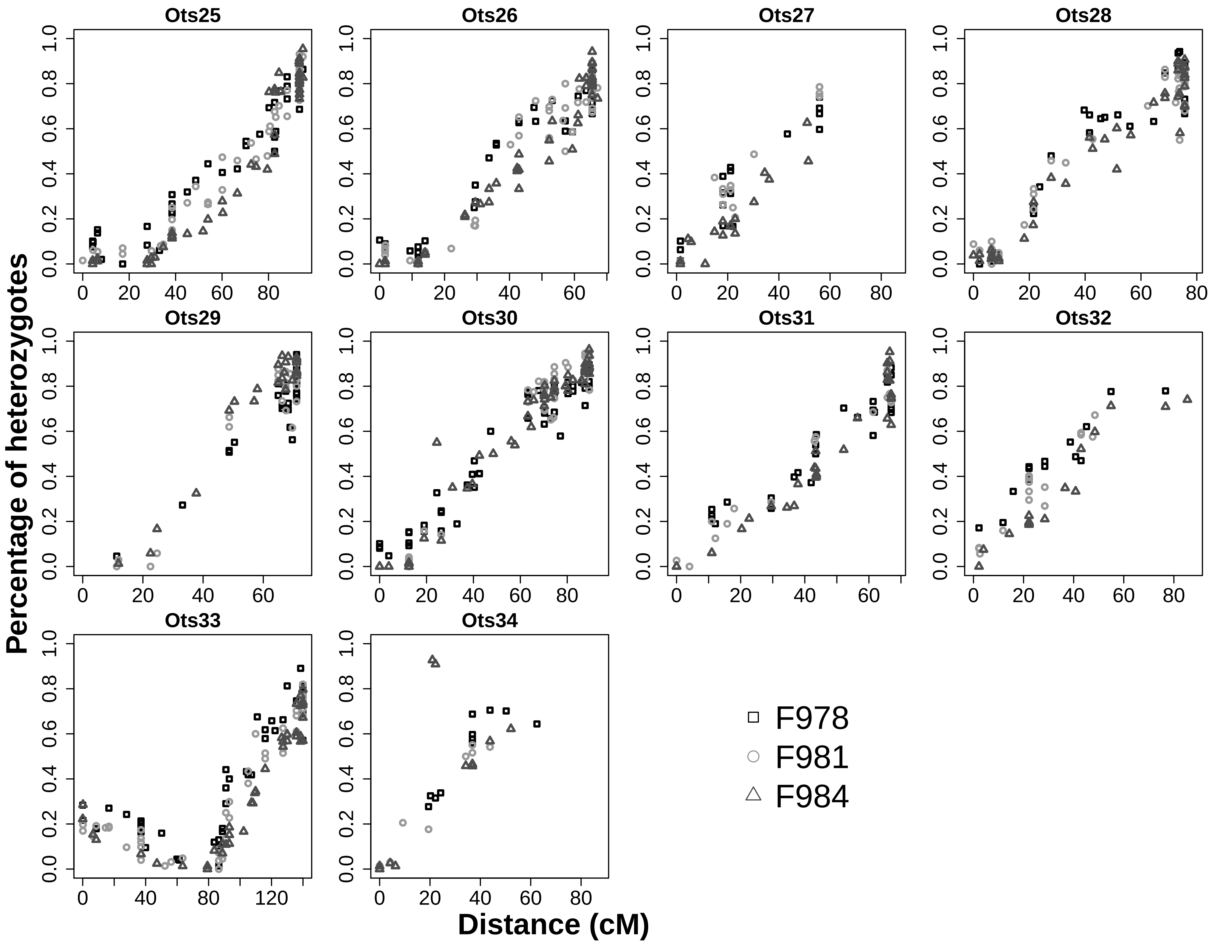
Ots25
Ots26
Ots27
Ots28
Ots29
Ots30
Ots31
Ots32
Ots33
Ots34
Percentage of heterozygotes
Distance (cM)
F978
F981
F984
